# Immunological Cross-Reactivity and Neutralisation of European Viper Venoms with the Monospecific *Vipera* *berus* Antivenom ViperaTAb

**DOI:** 10.3390/toxins6082471

**Published:** 2014-08-19

**Authors:** Nicholas R. Casewell, Ibrahim Al-Abdulla, David Smith, Ruth Coxon, John Landon

**Affiliations:** 1 MicroPharm Limited, Station Road Industrial Estate, Newcastle Emlyn, Carmarthenshire SA38 9BY, UK; E-Mails: i.abdulla@micropharm.co.uk (I.A.-A.); david.smith@micropharm.co.uk (D.S.); recoxon@micropharm.co.uk (R.C.); enquiries@micropharm.co.uk (J.L.); 2 Alistair Reid Venom Research Unit, Liverpool School of Tropical Medicine, Pembroke Place, Liverpool L3 5QA, UK

**Keywords:** antivenom, snake, snakebite, Viperidae, European viper, antibodies

## Abstract

Medically important cases of snakebite in Europe are predominately caused by European vipers of the genus *Vipera*. The mainstay of snakebite therapy is polyclonal antibody therapy, referred to as antivenom. Here we investigate the capability of the monospecific *V*. *berus* antivenom, ViperaTAb^®^, to cross-react with, and neutralise lethality induced by, a variety of European vipers. Using ELISA and immunoblotting, we find that ViperaTAb^®^ antibodies recognise and bind to the majority of toxic components found in the venoms of the *Vipera* species tested at comparably high levels to those observed with *V. berus*. Using *in vivo* pre-clinical efficacy studies, we demonstrate that ViperaTAb^®^ effectively neutralises lethality induced by *V. berus*, *V. aspis*, *V. ammodytes* and *V. latastei* venoms and at much higher levels than those outlined by regulatory pharmacopoeial guidelines. Notably, venom neutralisation was found to be superior to (*V. berus*, *V. aspis* and *V. latastei*), or as equally effective as (*V. ammodytes*), the monospecific *V. ammodytes* “Zagreb antivenom”, which has long been successfully used for treating European snake envenomings. This study suggests that ViperaTAb^®^ may be a valuable therapeutic product for treating snakebite by a variety of European vipers found throughout the continent.

## 1. Introduction

All of the medically-important venomous snakes found in Europe are members of the family Viperidae (vipers), with the vast majority belonging to the genus *Vipera*. These snakes are broadly referred to as the “European vipers” and include species such as *V. berus* from north Europe (including the UK, Nordic countries, the Netherlands, Poland and Germany), *V. aspis* from south and west Europe (including France and Italy), *V. ammodytes* from south and east Europe (including northeast Italy and the Balkans), *V. latastei* from south-west Europe (Portugal and Spain) and *V. ursinii* from central and eastern Europe (parts of France, Italy and the Balkans). In addition, two medically-important species from different genera, *Macrovipera lebetina* and *Montivipera xanthina* are both found in parts of south-eastern Europe, such as Turkey and Greece. These two species are closely related to the *Vipera* European vipers and until recently were classified as members of the same genus.

Snakebite is classified by the World Health Organisation as a neglected tropical disease, with perhaps as many as 94,000 people dying each year worldwide as the result of snake envenomings [1]. The majority of these cases occur in the tropical and sub-tropical regions of the world, inhabited by the rural poor [1,2]. However, recent estimates suggest that ~8,000 cases of snakebite occur each year in Europe, with 1,000 of these resulting in systemic envenoming and approximately four deaths [3]. Systemic envenoming by European vipers can cause severe pathology in humans, although fatalities are rare. The clinical manifestations can be variable and diverse in nature and this variability is observed across bites by different members of this genus (cf. [4,5,6]). Symptoms can include pain at the bite site, progressive local swelling, vomiting, tachycardia, hypotension, acute renal failure, haemorrhage, angio-oedema, pulmonary oedema, cardiac arrest, and on rare occasions, neurotoxicity and hypertension [4,5,6,7,8,9,10,11,12,13,14,15].

The mainstay of snakebite therapy consists of polyclonal antibodies made by hyper-immunising horses or sheep with relevant snake venom(s) and is termed antivenom. ViperaTAb^®^ is a monospecific ovine antivenom raised against the venom of *V. berus* and is manufactured by MicroPharm Limited in the United Kingdom. The antivenom is made from hyper-immunised sheep serum and consists of ovine Fab fragments which are cleaved from intact IgG molecules during manufacturing [16]. Subsequently, the Fab fragments are affinity purified using column chromatography, meaning that all of the antibodies present in ViperaTAb^®^ are specific to snake venom toxins. This is unlike a number of other snake antivenoms, where perhaps only 10% of the immunoglobulins present are actually specific to venom immunogens [17] due to the animals being immunised generating antibodies to other environmental antigens they are exposed to. ViperaTAb^®^ is formulated at a concentration of 25 mg/mL with a fill volume of 4 mL, resulting in 100 mg of specific Fab being delivered per therapeutic dose.

Fab antibodies offer a number of advantages over IgG and F(ab')_2_ antivenoms, most notably a pharmacokinetic advantage in that the molecular weight of Fab (~50 kDa) is much smaller than IgG (~150 kDa) and F(ab')_2_ (~100 kDa) and therefore permits a larger volume of distribution [18,19]. Considering the size of Fab is comparable to many of the toxic constituents of snake venoms (typically up to ~75 kDa in size), it is advantageous to have antibodies that are likely to have a similar volume of distribution to the toxins that they are targeting, as this may enable the earlier neutralisation of venom. However, these advantages come at some cost—whilst the faster elimination of Fab is likely to reduce the risk of longer-term adverse effects (*i.e*., serum sickness), careful clinical management is essential because of the risk of recrudescence if venom continues to diffuse out from the bite site after the Fab has been cleared. Nonetheless, due to the relatively small amounts of venom injected by *V. berus* during envenomings (~5–10 mg), recrudescence has not proven to be a major issue with the management of European viper snakebite, unlike observed elsewhere [20]. Indeed, a clinical trial undertaken with 231 patients in Scandinavia revealed that, of those envenomed victims that received antivenom, only 13% required a second vial of ViperaTAb^®^ during treatment [5]. Most importantly, ViperaTAb^®^ has been demonstrated to be highly efficacious and safe in human patients suffering systemic envenoming by *V. berus* in Scandinavia and this antivenom has been successfully used in the region for more than two decades [5,21].

Because of the inherent compositional variability of the toxic components found in the venom of different snake species [22,23,24], predicting the cross-neutralisation of heterologous venoms by monospecific antivenoms is exceedingly problematic [17,25,26]. Broad characterisations of the composition of *Vipera* venoms have yet to be undertaken for most species, although proteomic studies of *V. raddei* and members of the related genus *Macrovipera* revealed the presence of typical viperid toxin families, such as snake venom metalloproteinases, serine proteases, phospholipase A_2_, C-type lectins and disintegrins [27,28]. Specific toxic components have also been isolated and characterised from *Vipera* venoms, including those involved in coagulopathy and neurotoxicity [14,29,30,31]. Importantly, simple protein profiles of different *Vipera* venoms suggest that there are broad similarities in toxin composition [32], whilst evidence that the monospecific equine F(ab')_2_ *V. ammodytes* “Zagreb antivenom” (formally named “viper venom antiserum, European (equine)”, Institute of Immunology, Zagreb, Croatia) is capable of neutralising the lethal effects of *V. berus* and *V. aspis*, in addition to *V. ammodytes* [8,12,13,33], reinforces the suggestion of toxin conservation.

Here we investigate the immunological cross-reactivity and pre-clinical neutralisation of a variety of European vipers with the monospecific *V. berus* antivenom ViperaTAb^®^. We first assess the immunological cross-reactivity of ViperaTAb^®^ antibodies to a range of European viper venoms, using ELISA and immunoblotting. We next use a murine *in vivo* pre-clinical assay, which is the gold-standard for predicting antivenom efficacy, to test the neutralising potency of ViperaTAb^®^ against four representative *Vipera* venoms (*V. berus*, *V. ammodytes*, *V. aspis* and *V. latastei*). Finally, we assess the resulting cross-reactivity data generated using ViperaTAb^®^ with results gained in these assays using the historically well-used Zagreb antivenom as a comparator.

## 2. Results and Discussion

### 2.1. Immunological Cross-Reactivity Using ELISA

We first assessed the ability of the monospecific *V. berus* antivenom ViperaTAb^®^ and the monospecific *V. ammodytes* Zagreb antivenom to recognise and bind to the toxins present in a range of European viper venoms. We used the ELISA technique to quantify the relative binding capacity of each antivenom to the venom from each of the following species: *V. aspis* (France), *V. ammodytes* (Croatia), *V. berus* (Russia), *V. latastei* (Spain), *V. ursinii* (Russia), *M. lebetina* (Azerbaijan) and *M. xanthina* (Turkey).

**Figure 1:**
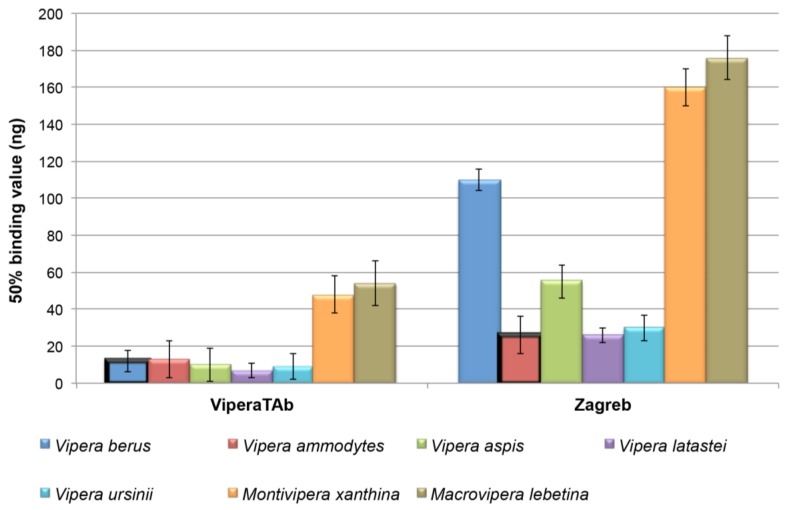
*In vitro* ELISA cross-reactivity of ViperaTAb^®^ and Zagreb antivenoms to a variety of European viper venoms. The 50% binding value represents the amount of antivenom (in ng) required to bind half of the test venom on the microtitre plate in the ELISA experiment. Thus, the lower the 50% value, the greater the binding capacity of the antivenom. Bars for the venoms used to raise the antibodies present in each antivenom are highlighted by bold borders (*V. berus* for ViperaTAb^®^; *V. ammodytes* for Zagreb). Error bars represent standard deviation.

Both antivenoms were found to recognise all of the test European viper venoms screened, albeit to varying extents (Figure 1). ViperaTAb^®^ displayed high levels of binding (indicated by the lowest 50% binding values) to the venoms of *V. latastei*, *V. ammodytes*, *V. aspis* and *V. ursinii*, with the binding levels detected comparable to that of the venom used during antivenom production (*V. berus*) (Figure 1). These results suggest that the antibodies present in ViperaTAb^®^ antivenom are capable of extensive recognition and cross-reactivity with the toxic components present in the venom of other European viper species.

A greater extent of variation in cross-reactivity was observed with the Zagreb product, although of the venoms described, only that from *V. berus* was found to cross-react poorly. The venoms of *V. latastei* and *V. ursinii* bound at a comparable level to that of the venom used for immunisation (*V. ammodytes*) (Figure 1). Note that the results obtained for the two antivenoms are not directly comparable to each other, as different secondary antibody preparations (anti-sheep antibodies for ViperaTAb^®^ and anti-horse antibodies for Zagreb) were required to quantify the level of venom-antibody binding. Nonetheless, venom from *M. xanthina* and *M. lebetina* cross-reacted with both antivenoms to a much lower extent than observed with the venoms from the different *Vipera* species (Figure 1), suggesting that substantial inter-generic differences in toxin composition may exist between these different genera of European vipers. However, we cannot exclude the possibility that the different venoms tested may vary in their ability to bind to the ELISA plates used, and that this confounding factor could potentially explain some of the variation in venom-antivenom binding noted, particularly when relating to the results obtained from the non-*Vipera* species.

### 2.2. Immunological Cross-Reactivity Using Immunoblotting

**Figure 2:**
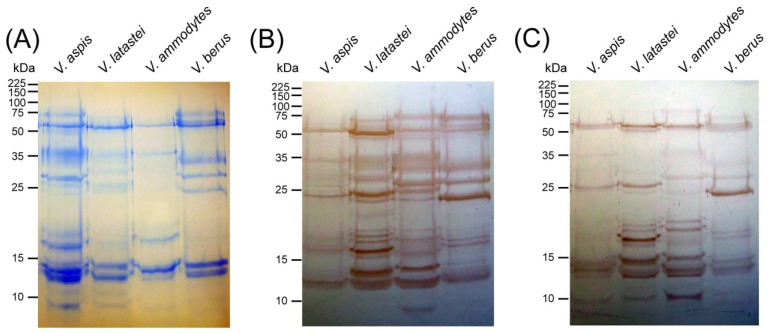
The protein profiles of four *Vipera* venoms and their immunological cross-reactivity with ViperaTAb^®^ and Zagreb antivenoms. (**A**) One dimensional SDS-PAGE gel of four *Vipera* venoms stained with Coomassie Blue R-250 and their immunological cross-reactivity with (**B**) the monospecific *V. berus* antivenom ViperaTAb^®^ and (**C**) the monospecific *V. ammodytes* Zagreb antivenom.

We next visualised venom-protein antibody-binding by using immunoblotting experiments with the venoms of four representative Western European vipers (*V. aspis*, *V. ammodytes*, *V. berus* and *V. latastei*) and the two antivenoms. We first characterised the protein profiles of the four venoms by one-dimensional SDS-PAGE gel electrophoresis (Figure 2A). The venom composition of the four *Vipera* species exhibit a wide range of molecular weight proteins, from ~10 kDa to ~75 kDa in size. Notably, a number of protein bands appear to be conserved in the venom of the four species, although there also appears to be some degree of inter-specific variation in toxin composition, with *V. aspis* containing the most complex profile and *V. ammodytes* the most simple (Figure 2A).

Immunoblotting these separated venom proteins with ViperaTAb^®^ antivenom revealed extensive immunological cross-reactivity, with the antibodies recognising the majority of proteins present in the gel (Figure 2B). In the case of *V. ammodytes* and *V. latastei*, the binding of ViperaTAb^®^ antivenom to venom toxins revealed a number of toxin constituents that were not readily apparent in the initial protein profiles. However, the intensity of binding to *V. aspis* venom was notably lower than that observed with the other three *Vipera* species. Control experiments using normal sheep serum (sourced from non-immunised animals) as a positive control and secondary antibodies only as a negative control, revealed no evidence of immunological cross-reactivity with venom proteins.

Immunoblotting with Zagreb antivenom revealed a similar pattern as that observed with ViperaTAb^®^, with many of the venom proteins evident in the gel being recognised by the antivenom antibodies (Figure 2C). As with ViperaTAb^®^, the intensity of venom-antibody binding was lower for *V*. *aspis* than the other members of the genus. For the same reasons as described for the ELISA experiments, the intensity of antibody binding observed in the immunoblots is not directly comparable between the two antivenoms. However, it is readily apparent that considerably more venom proteins were recognised by ViperaTAb^®^ than by the Zagreb antivenom (Figure 2B,C) suggesting that *V*. *berus* venom, which was used during the immunisation process, may contain a more representative venom composition for the genus than that of *V. ammodytes*.

### 2.3. In Vivo Neutralisation of Venom Lethality

**Figure 3:**
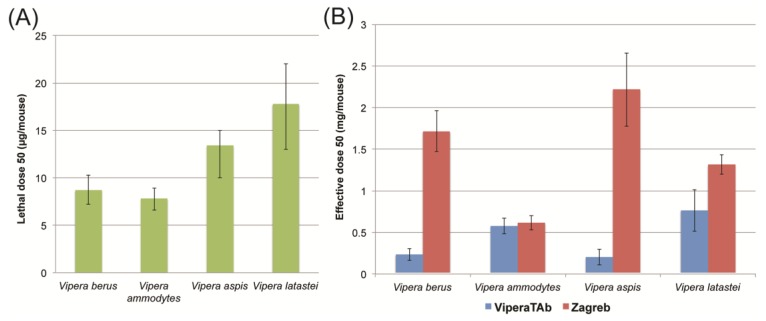
Relative *in vivo* potencies of ViperaTAb^®^ and Zagreb antivenoms against venoms from four species of the genus *Vipera.* (**A**) Lethal dose 50 (LD_50_) values for each of the four *Vipera* venoms studied; (**B**) Effective dose 50 (ED_50_) values detailing the relative potency of ViperaTAb^®^ and Zagreb antivenoms. The effective dose is the amount of antivenom antibodies required to protect 50% of a population of mice from the lethal effects of five LD_50_ doses of each venom. Error bars represent 95% confidence intervals.

Whilst evidence of extensive immunological cross-reactivity appears to be a prerequisite for ensuring antivenom efficacy, evidence of antivenom-venom binding does not guarantee that the toxin components present in venom will be neutralised *in vivo* (e.g., [17]). The gold-standard predictor for antivenom efficacy is the murine effective dose 50 assay (ED_50_), which is a World Health Organisation approved assay to determine the amount of antivenom required to protect half a mouse population from the lethal effects of venom. First we determined the lethal dose 50 (LD_50_) dose of each venom (Figure 3A), before delivering five LD_50_ doses of venom that had been mixed with varying doses of antivenom in to groups of mice to determine: (I) whether the antivenom protects against lethality and (II) if so, at what dose. We undertook these experiments using venoms from the four Western European vipers analysed in the immunoblotting assays (*V. aspis*, *V. ammodytes*, *V. berus* and *V. latastei*) and both ViperaTAb^®^ and Zagreb antivenoms.

Both antivenoms were found to effectively neutralise five times the venom LD_50_ from *V. berus*, *V. ammodytes*, *V. aspis* and *V. latastei* (Figure 3). These representative venoms cover a broad geographical distribution of *Vipera* snakes, namely north, south-east, west and south-west Europe, respectively. Unlike the immunological cross-reactivity data, the *in vivo* ED_50_ data provides a direct comparison of the efficacy of the two antivenoms. For all four venoms tested, a smaller amount of ViperaTAb^®^ antivenom was required to prevent murine lethality, and therefore achieve complete protection (Figure 3). This is evident to the greatest extent when neutralising *V. aspis* and *V. berus* venoms, where eleven and seven times the amount of Zagreb antivenom is required to prevent lethality than observed with ViperaTAb^®^, respectively. The effect is, as expected, less dramatic when comparing the neutralisation potencies of the two antivenoms against the venom of *V. ammodytes*, as this is the venom immunogen used to manufacture the Zagreb product (Figure 3). However, these results indicate that ViperaTAb^®^ is at least as equally effective as the Zagreb product at neutralising the venom of *V. ammodytes*, despite it not being used as an immunogen during antibody production.

The ED_50_ values displayed in Figure 3 can be translated into “protective units”, which are defined as “the number of LD_50_ neutralised per mL of antivenom” by using the British Pharmacopoeial test method. From these calculations, the potency of ViperaTAb^®^ can be compared with the Pharmacopoeial minimum standard set for European viper antivenoms (Table 1). Notably, in all cases, ViperaTAb^®^ provides potency results that greatly exceed (by at least double) the required minimum set for effective European viper antivenoms.

**Table 1:** ViperaTAb^®^ *in vivo* potency results compared to the regulatory Pharmacopoeial standards.

Venom	Pharmacopoeial minimum specification	ViperaTAb^®^ antivenom
*Vipera berus*	50	597
*Vipera ammodytes*	100	222
*Vipera aspis*	100	690
*Vipera latastei*	N/A	176

Data is expressed as “protective units”, which are defined as the number of LD_50_ venom doses neutralised per mL of antivenom. N/A, no specification for *V. latastei* is provided in the Pharmacopoeial guidelines.

### 2.4. Discussion

Both *in vitro* antivenom binding experiments demonstrate the inherent cross-reactivity of a variety of European viper venoms to the two monospecific antivenoms, ViperaTAb^®^ and Zagreb. The ELISA assay demonstrates that a wide range of *Vipera* venoms are recognised by ViperaTAb^®^ with similar binding levels to those observed for *V. berus*, which was used as the immunogen. ViperaTAb^®^ was also capable of binding to the venom of non-*Vipera* species (*M. xanthina* and *M. lebetina*), although the level of binding observed was reduced when compared to the *Vipera* species—this same pattern was also found with the anti-*V. ammodytes* Zagreb antivenom. Therefore the effectiveness of both ViperaTAb^®^ and Zagreb antivenoms for the treatment of snakebite against non-*Vipera* species remains unclear at this time, but it appears that these antivenoms are likely to be less effective due to reduced levels of antibody-venom binding (Figure 1).

The immunoblotting experiments permit the visualisation of the cross-reactivity detected by ELISA. Notably, both antivenoms recognise a broad molecular weight range of venom components present in the venoms of the four *Vipera* species, thereby validating the results produced by ELISA. The only notable exceptions are (I) that the decrease in binding by the Zagreb antivenom to *V. berus* venom observed in the ELISA experiment (Figure 1) does not appear to be apparent in the immunoblot, as the intensity and range of venom protein binding is comparable to the other venoms and the toxin components found in the SDS-PAGE gel (Figure 2), and (II) evidence that ViperaTAb^®^ does not appear to bind to *V. aspis* venom proteins with the same intensity as to the other venoms in the immunoblot (Figure 2), despite comparable binding levels observed by ELISA (Figure 1).

In contrast to simply providing an indication of whether antibodies recognise and bind to venom components, the *in vivo* pre-clinical ED_50_ assay provides the most robust evidence (outside of clinical use in humans) of antivenom efficacy, by assessing neutralisation of venom-induced mortality in an animal model. Notably, our results suggest that ViperaTAb^®^ is likely to be highly efficacious for the treatment of snake envenoming by a number of *Vipera* species, in addition to *V. berus* (Figure 3, Table 1). Since ED_50_ assays are specified as efficacy release criteria for European antivenoms, the results presented here may be used to justify the potential use of ViperaTAb^®^ in clinical trials to treat human envenomings by *V. aspis*, *V. latastei* and *V. ammodytes*. The extent of pre-clinical neutralisation well exceeded the minimum potency levels defined by Pharmacopoeial guidelines in all cases, and the pre-clinical efficacy observed for *V. berus*, which is comparable to the other three species, has already been demonstrated to translate in to clinical efficacy through the decades of successful use of ViperaTAb^®^ to treat human patients in Scandinavia [5,21].

The results are perhaps more pertinent when comparing the ED_50_ potencies of ViperaTAb^®^ with the Zagreb antivenom. The Zagreb product has been successfully used to treat systemic envenoming by bites by *V. ammodytes*, *V. aspis* and *V. berus* in Europe for many years [8,12,13,33]. However, the pre-clinical data presented here suggests that ViperaTAb^®^ outperforms the Zagreb antivenom by preventing lethality induced by *V. berus*, *V. aspis* and *V. latastei* venom at much lower doses, whilst comparable results were observed with *V. ammodytes* venom. Despite the absence of pre-clinical data for *V. ursinii*, the results of the immunological cross-reactivity study suggest that ViperaTAb^®^ may also have clinical potential for the treatment of bites by this species, although ED_50_ assays will be required to rigorously test this hypothesis.

The differences in pre-clinical potency observed here between the two antivenoms may in part reflect the fact that ViperaTab^®^ is an affinity-purified antivenom, and therefore, milligram to milligram, contains a higher number of antibodies specific to *Vipera* venom proteins than that of the non-purified Zagreb product. Ultimately, we therefore conclude that the highly efficacious anti-*V. berus* antivenom ViperaTAb^®^ may also be an effective treatment for snakebite caused by a number of different *Vipera* species found across Europe.

## 3. Experimental Section

### 3.1. Materials

Venoms used in this study were purchased from Latoxan, Valence, France. ViperaTAb^®^ antivenom was provided by MicroPharm Limited, Carmarthenshire, UK and consists of 25 mg/mL of ovine affinity-purified Fab antibodies specific to the venom of *V. berus*. Zagreb antivenom was purchased from the Institute of Immunology, Zagreb, Croatia and consists of ~100 mg/mL of equine of total F(ab')_2_ antibodies from horses that had been immunised with the venom of *V. ammodytes*.

### 3.2. ELISA

Ninety-six well microtitre plates were coated with each venom dissolved in coating buffer (0.2 µg/well; sodium bicarbonate buffer 0.1 M, pH 9.6 containing 0.1% thiomerosal; 2 h at 37 °C). Coated plates were washed three times with washing buffer (300 µL; sodium phosphate 10 mm, sodium chloride 0.8%, potassium chloride 0.02%, thiomerosal 0.01% and Tween 20, 0.1%, pH 7.4) to remove unbound venom, and then blocked by incubation with the same buffer for 1h at 37 °C. One hundred microlitre doubling dilutions of the antivenoms (or a non-immune sheep or horse IgG control dissolved in washing buffer) were incubated with the venom for 1 h at 37 °C, before washing as before. Bound antibodies were visualised by adding commercially sourced (Sigma-Aldrich, St. Louis, MO, USA) secondary antibodies (donkey anti-sheep for ViperaTAb^®^ experiments; sheep anti-horse for Zagreb experiments; both coupled to horseradish peroxidase and diluted 1:500 in washing buffer, 100 µL/well) and incubated for 1 h at 37 °C. After washing a third time, substrate solution was added (100 µL/well of o-phenylenediamine, 0.01%, dissolved in sodium citrate buffer, 0.07 M, pH 5.0 containing H_2_O_2_ 20 µL/100 mL of a 30% *v*/*v* solution) and the reaction stopped after 10 min with the addition of sulphuric acid (3 M, 50 µL/well). The optical density of the solution was read at 492 nm using an automatic 96 well ELISA plate reader. The amount of antivenom sufficient to bind 50% of the venom was calculated from a graph of optical density plotted against antivenom concentration.

### 3.3. SDS-PAGE and Immunoblotting

Venoms from the four Western European *Vipera* species (*V. ammodytes*, *V. aspis*, *V. berus* and *V. latastei*) were reconstituted to 1 mg/mL in reduced protein loading buffer containing 2-mecaptoethanol and boiled for 10 min to facilitate protein unfolding. Seven µg of venom, together with molecular weight markers (Broad Range Molecular Weight Protein Marker, Promega, Southampton, UK), were added to a 15% SDS-PAGE gel and fractionated under 200 volts, as previously described [17]. The resultant separated proteins were visualised by staining with Coomassie Blue R-250.

Immunoblotting experiments were carried out in an identical manner except that after the completion of protein separation, the gels were electro-blotted onto 0.45 µm nitrocellulose membranes at 100 volts, using the manufacturer’s protocols (Bio-Rad, Hemel Hempstead, UK). Following confirmation of successful protein transfer by reversible Ponceau S staining, the membranes were incubated overnight in blocking buffer (5% non-fat milk in TBST buffer (0.01 M Tris-HCl, pH 8.5, 0.15 M NaCl and 1% Tween-20)), followed by six washes of TBST over 90 min and incubation overnight with either ViperaTAb^®^ or Zagreb antivenoms diluted 1:5000 in blocking buffer. The immunoblots were washed again as described above and then incubated for two hours with horseradish peroxidise-conjugated donkey anti-sheep secondary antibody (ViperaTAb^®^) or horseradish peroxidise-conjugated sheep anti-horse secondary antibody (Zagreb) diluted 1:2000 in TBST. The immunoblots were then washed again and venom-protein antivenom-antibody binding visualised with the addition of DAB substrate (50 mg 3,3-diaminobenzidine, 100 mL PBS and 0.024% hydrogen peroxide; Sigma-Aldrich, St. Louis, MO, USA).

### 3.4. In Vivo Venom Neutralisation (ED_50_)

The murine ED_50_ assay determines the amount of antivenom required to protect half a mouse population from the lethal effects of a venom. These assays were undertaken as previously described [17,26] by intravenously injecting five groups of five male TSW mice (18–20 g) with varying doses of each antivenom, which had previously been mixed and incubated at 37 °C with five lethal doses (LD_50_) of the test venom. The potency of the antivenom was calculated from the number of mice surviving after 24 h using probit analysis [34]. Venom LD_50_ doses, the amount of venom required to kill 50% of mice, were previously determined by injecting five groups of five mice with varying doses of venom and calculating the LD_50_ from mortality numbers using probit analysis, as previously described [17,26]. For ethical reasons, ED_50_ assays were only undertaken to assess and compare the clinical efficacy of ViperaTAb^®^ and Zagreb antivenoms against venom from four representative species of European viper (*V. berus*, *V. ammodytes*, *V. aspis* and *V. latastei*). All animal work was undertaken with local ethical approval and with licensed approval from the UK Home Office.

## 4. Conclusions

Immunological experiments revealed that the anti-*V. berus* antivenom, ViperaTAb^®^, exhibits substantial cross-reactivity with the venoms of other *Vipera* snake species. Notably, ViperaTAb^®^ neutralised the lethality induced by *V. berus*, *V. aspis*, *V. ammodytes* and *V. latastei* venoms *in vivo* and at much higher levels than those outlined by regulatory pharmacopoeial guidelines. When compared with the existing, widely used, anti-*V. ammodytes* “Zagreb” antivenom, ViperaTAb performed favourably, with venom neutralisation found to be superior (*V. berus*, *V. aspis* and *V. latastei*), or as equally effective (*V. ammodytes*). Our results suggest that, in addition to *V. berus*, ViperaTAb^®^ shows clinical promise for treating snakebite by a number of other European vipers found throughout the continent.
